# Dual inhibiting OCT4 and AKT potently suppresses the propagation of human cancer cells

**DOI:** 10.1038/srep46246

**Published:** 2017-04-06

**Authors:** Wenxin Li, Yanwen Zhou, Xiaoqian Zhang, Ying Yang, Songsong Dan, Tong Su, Shiqi She, Weilai Dong, Qingwei Zhao, Jia Jia, Hangping Yao, Min Zheng, Bo Kang, Ying-Jie Wang

**Affiliations:** 1 State Key Laboratory for Diagnosis and Treatment of Infectious Diseases, Collaborative Innovation Center for Diagnosis and Treatment of Infectious Diseases, the First Affiliated Hospital, School of Medicine, Zhejiang University, Hangzhou, Zhejiang 310003, China; 2 College of Life Sciences, Zhejiang University, Hangzhou, Zhejiang 310058, China; 3 Department of Pharmacy, the First Affiliated Hospital, School of Medicine, Zhejiang University, Hangzhou, Zhejiang 310003, China; 4 Shanghai Center for Bioinformation Technology, Shanghai 201203, China

## Abstract

AKT serves as an epigenetic modulator that links epigenetic regulation to cell survival and proliferation while the epigenetic mediator OCT4 critically controls stem cell pluripotency and self-renewal. Emerging evidence indicated their complicated interplays in cancer cells and cancer stem cells (CSCs), and inhibiting either one may activate the other. Thus, in this study, we propose a strategy to targeting both factors simultaneously. Firstly, a combination of an OCT4-specific shRNA and the specific AKT inhibitor Akti-1/2 potently suppressed the propagation of human embryonal carcinoma cells, adherent cancer cells and stem-like cancer cells, establishing the proof-of-concept that dual inhibiting OCT4 and AKT can effectively target various cancer cells. Next, we combined Akti-1/2 with metformin, a widely-prescribed drug for treating type 2 diabetes, which was reported to down-regulate OCT4 expression. The metformin + Akti-1/2 combo significantly altered multiple signaling and epigenetic pathways, induced growth arrest and cell death of adherent and stem-like glioblastoma U87 cells, and attenuated their tumorigenicity *in vivo*. Taken together, we demonstrate here that simultaneously targeting an epigenetic mediator and an epigenetic modulator, by dual inhibiting OCT4 and AKT, can have significantly improved efficacies over single treatment in suppressing the propagation of CSCs as well as the entire bulk of differentiated cancer cells.

It has long been proposed that the clonogenic growth and self-renewal potential of cancer cells are restricted to a subset of cells within tumors known as cancer stem cells (CSCs) or stem-like cancer cells^1^,^2^. The CSCs that can functionally initiate a tumor upon transplantation are also termed as tumor-initiating cells^2^. CSCs are thought to critically account for tumor heterogeneity, metastasis, and resistance to anticancer therapies^3^. A series of recent studies obtained direct experimental evidence that genetic mutation of tissue stem cells is an important determinant of cancer risk^4^,^5^, rendering strong support for the CSC model.

While little is known about the molecular mechanisms underlying the transition from normal tissue stem cells to CSCs, it is increasingly apparent that CSCs are highly plastic and there can be dynamic interconversions between CSC-like and non-CSC-like cancer cells under certain conditions, and epigenetic regulation plays a pivotal role in controlling the plasticity of cancer cells^2^,^3^,^6^. For example, the histone H3K4 demethylase JARID1B was shown to be reversibly expressed in melanoma cell lines that was associated with an interconversion between slow-cycling, CSC-like and rapid-cycling, non-CSC-like phenotypes^7^. Recently, Feinberg *et al*.^8^ proposed a framework for better understanding cancer epigenetics. Based on their roles in regulating cancer epigenetics and their contribution to malignancy, three classes of genes were defined: the epigenetic modulators, the epigenetic modifiers, and the epigenetic mediators (Fig. S1). The epigenetic modulators sense and integrate environmental signals and transduce them onto their downstream targets that include the modifiers and the mediators. The modifiers consist of all components of epigenetic machinery such as those involved in regulating DNA methylation, histone modification and chromatin organization. The mediators largely overlap with genes responsible for regulating stem cell self-renewal and differentiation and driving cancer cells toward a more stem-like state. Epigenetic modulators usually regulate the modifiers and mediators via direct protein-protein interaction and various post-translational modifications (PTMs), while the modifiers dictate the epigenetic status of the mediator genes, altering their transcription and post-transcriptional processing. It is noteworthy that besides the “modulator-modifier-mediator” top-down regulation, the mediators can regulate the transcription of the modifiers and modulators in a feedback manner^8^.

Among the epigenetic modulators, AKT links epigenetic regulation to cell survival and proliferation. It can phosphorylate a number of key chromatin-modifying enzymes, such as the histone acetyltransferase p300, the DNA methyltransferase DNMT1, the H3K27 methyltransferase EZH2, and the histone ubiquitylating enzyme BMI1, altering the function of these enzymes^9^. Besides its well established roles in anti-apoptosis and cell survival, emerging evidence indicated that AKT is also a key player in stemness regulation and CSC biology, and thus a promising target for developing anticancer drugs against CSCs^10^,^11^. OCT4, a master transcription factor controlling stem cell self-renewal and pluripotency, is considered as one of the most important mediators for cancer epigenetics^8^. Although somewhat controversial, the presence of OCT4 mRNA in human cancer tissues and cell lines and its enrichment in CSCs have been supported by a wealth of literature, and the participation of OCT4 in various CSC functions such as its self-renewal and survival, epithelial-mesenchymal transition (EMT) and metastasis, and drug resistance development is implicated from considerable OCT4 knockdown and overexpression-based studies^12^.

Importantly, there are cross-talks between AKT and OCT4 at multiple levels^13^. In embryonic stem cells (ESCs), AKT can indirectly regulate the mRNA level, protein stability, and transcriptional activity of OCT4 via a number of pathways such as the AKT/GSK3β/β-catenin pathway and the AKT/SMAD2/3/NANOG pathway. Reversely, OCT4-to-AKT connection is mediated via TCL1, CD49f, and other proteins. Thus, these two master regulators can form an AKT-OCT4 regulatory circuit in ESCs, controlling cell survival and self-renewal^13^. Furthermore, OCT4 has been demonstrated as a direct substrate of AKT in mESCs^14^, hESCs^15^, mouse embryonal carcinoma cells (ECCs)^16^, human ECCs^17^,^18^, and human CSCs^18^,^19^, and AKT-phosphorylated OCT4 can regulate the transcription of AKT1 in a positive feedback manner to over-activate the AKT-OCT4 regulatory circuit and thereby promoting the anti-apoptosis pathways and survival of hECCs^17^. Depending on the cellular contexts, inhibiting OCT4 or AKT alone may either activate or inactivate its counterpart, resulting in considerable uncertainties in therapeutic efficacies. Therefore, in this study, we set out to test if simultaneously targeting an epigenetic modulator and an epigenetic mediator, by dual inhibiting OCT4 and AKT, could have significantly improved effects over single treatment in dampening the self-renewal and propagation of CSCs as well as the entire bulk of cancer cells.

## Results

### OCT4 shRNA + Akti-1/2 combo effectively suppresses the propagation of NCCIT cells

**Figure 1:**
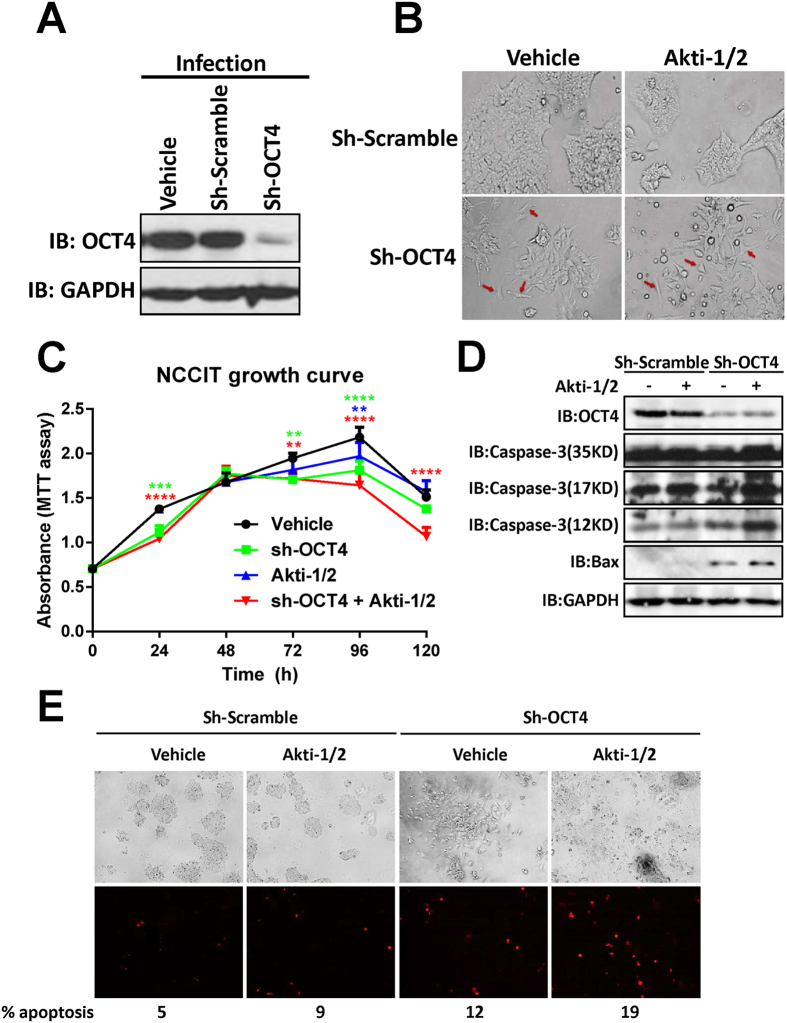
OCT4 shRNA + Akti-1/2 suppresses the propagation of NCCIT cells and triggers their apoptosis. (**A**) NCCIT cells grown in 6-well plates were infected with culture medium only (vehicle), lentiviruses harboring scramble shRNA (sh-Scramble) or OCT4 shRNA (sh-OCT4). 3 days post infection, cells were harvested and lysed, and the whole cell lysates were subjected to immunoblotting with the indicated antibodies. **(B)** NCCIT cells were infected with sh-Scramble or sh-OCT4, and simultaneously treated with DMSO (vehicle) or 10 μM Akti-1/2 for 3 days. The images were captured under an Olympus IX81 microscope at 200X magnification. Red arrows indicate differentiated cells. **(C)** NCCIT cells grown in 96-well plates were infected with sh-OCT4 and simultaneously treated with DMSO (vehicle) or 10 μM Akti-1/2, and subjected to MTT assay at each time point. Data were expressed as mean ± SD of triplicate measurements from one of three independent experiments which gave similar results. The colored asterisks indicate the difference between each treatment group and vehicle group by significance levels (*p < 0.05, **p < 0.01, ***p < 0.001, ****p < 0.0001). **(D)** NCCIT cells grown in 6-well plates were treated in the same manner as (**B**) 3 days post infection, cells were harvested and lysed, and the whole cell lysates were subjected to immunoblotting with the indicated antibodies. **(E)** Adherent live NCCIT cells grown in 6-well plates were stained with 5 μg/ml Propidium Iodide for 10 min at 37 °C. Cells were examined using an Olympus fluorescence microscope at the excitation wavelength of 535 nm, the total cells and the red fluorescent cells that represent late phase apoptotic cells were counted in captured images (40X magnification), and the percentages of apoptotic cells were given at the bottom of the images.

To assess the effect of dual inhibiting AKT and OCT4, human embryonal carcinoma NCCIT cells were first infected with an OCT4 shRNA lentivirus (sh-OCT4), followed by Akti-1/2 treatment. Consistent with our previous result^17^, OCT4 shRNA reduced the OCT4 protein level by greater than 90% (Fig. 1A), leading to the disappearance of cell colonies and appearance of differentiated cells with an elongated cell shape (Fig. 1B, red arrows). Treating the scramble shRNA-infected cells with 10 μM Akti-1/2, a specific inhibitor of AKT, for 3 days, only caused minor changes in cell and colony morphology, while sh-OCT4-infected cells subject to Akti-1/2 treatment exhibited dramatic morphological changes including complete disappearance of cell colonies, cell elongation, shrinkage, round up and detachment (Fig. 1B). MTT-based cell proliferation assay showed that starting from day 3, there were increasing differences in total cell numbers among the four groups, and the “sh-OCT4 + Akti-1/2” group had significantly lower cell numbers than other three groups at day 4 and day5 (Fig. 1C). To find out if the seen growth inhibition is partly due to apoptosis-induced cell death, the four groups of cells were collected and analyzed with immunoblotting for multiple apoptotic biomarkers. Interestingly, either sh-OCT4 or Akti-1/2 alone increased the protein levels of activated caspase-3 p17 and p12 fragments as well as Bax, and the combined treatment resulted in the highest levels of those apoptosis markers (Fig. 1D). Propidium Iodide (PI) staining for late phase apoptotic cells confirmed the highest percentage of apoptotic cells with the combined treatment (Fig. 1E). Thus, dual inhibiting OCT4 and AKT effectively suppressed the propagation of human embryonal carcinoma cells and triggered their apoptotic death.

### OCT4 shRNA + Akti-1/2 combo effectively suppresses the propagation of adherent cancer cells

**Figure 2:**
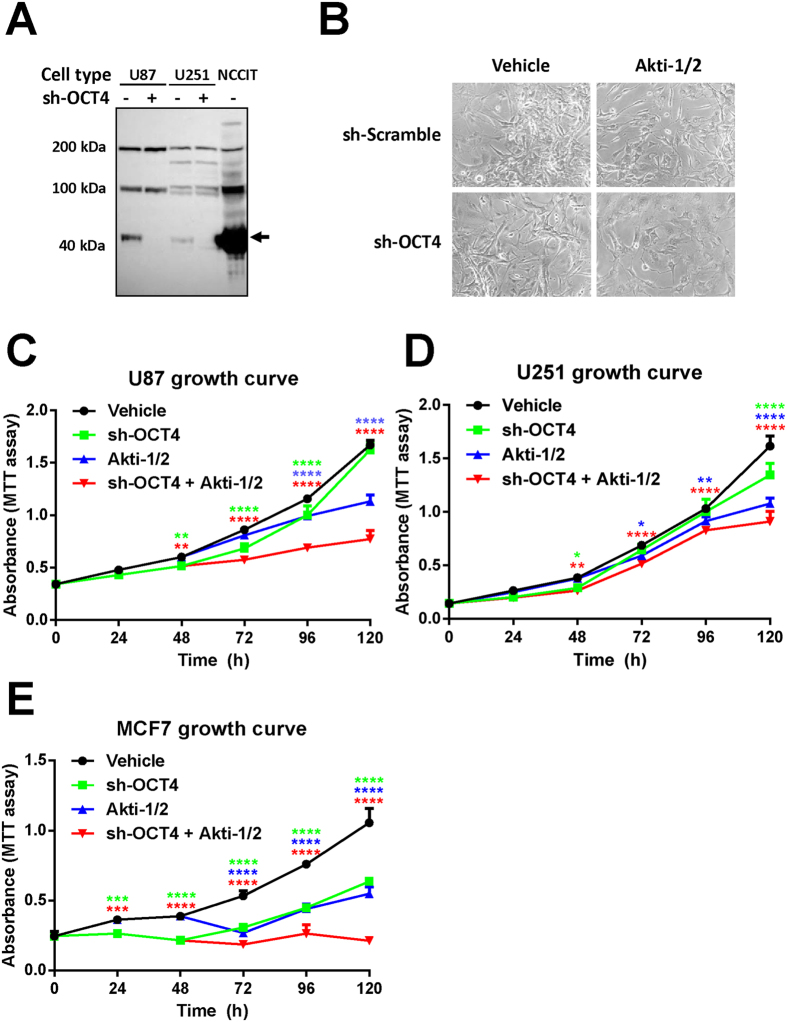
OCT4 shRNA + Akti-1/2 suppresses the propagation of adherent cancer cells. (**A**) U87, U251, NCCIT cells grown in 6-well plates were infected with lentiviruses harboring OCT4 shRNA (sh-OCT4) or culture medium only. 3 days post infection, cells were harvested and lysed, and the whole cell lysates were subjected to immunoblotting with an anti-OCT4. The amount of total NCCIT protein loaded was only 1/40 of that of U87 and U251 cells. (**B**) Adherent U87 cells were infected with sh-Scramble or sh-OCT4, and simultaneously treated with DMSO (vehicle) or 5 μM Akti-1/2 for 5 days. The images were captured under an Olympus IX81 microscope at 200X magnification. **(C–E)** Adherent U87 cells (**C**), U251 cells (**D**) or MCF7 cells (**E**) grown in 96-well plates were infected with sh-OCT4 and simultaneously treated with DMSO (vehicle) or 5 μM Akti-1/2, and subjected to MTT assay at each time point. Data were expressed as mean ± SD of triplicate measurements from one of three independent experiments which gave similar results. The colored asterisks indicate the difference between each treatment group and vehicle group by significance levels (*p < 0.05, **p < 0.01, ***p < 0.001, ****p < 0.0001).

To assess the effect of dual inhibiting OCT4 and AKT on cancer cells of somatic origin, we chose two human glioblastoma cell lines (U87 and U251) that are known to express low levels of OCT4^19^,^20^, infected them with sh-OCT4, and then treated them with Akti-1/2. As shown in Fig. 2A, although the expression levels of OCT4 in both glioblastoma cell lines were already much lower than that in NCCIT cells, sh-OCT4 further reduced their OCT4 levels dramatically (Fig. 2A). However, sh-OCT4 alone had no discernible effect on the morphology of adherent U87 cells (Fig. 2B) and the growth rate of U251 cells (Fig. 2D), and only marginally attenuated the propagation of U87 cells (Fig. 2C) and MCF-7 cells (Fig. 2E). Likewise, Akti-1/2 alone only had some effect on U87 cell morphology (Fig. 2B) and on the growth rate of three cell lines (Fig. 2C–E). Remarkably, dual treatment of all three cancer cell lines with sh-OCT4 + Akti-1/2 had significantly improved effects over single treatment in attenuating their propagation. Taken together, despite the fact that the endogenous OCT4 levels in somatic cancer cells were extremely low, and knocking down OCT4 alone had no prominent effects, combining sh-OCT4 with Akti-1/2 dramatically suppressed the propagation of adherent cancer cells.

### Metformin + Akti-1/2 combo alters multiple signaling and epigenetic pathways

The above studies established the proof-of-concept that dual inhibiting OCT4 and AKT can effectively block the propagation of human cancer cells. For potential clinical applications, it would be ideal if the sh-OCT4 can be replaced with a small-molecule compound that can specifically inhibit OCT4 expression. We have previously shown that one of the tryptophan metabolites, ITE, can suppress the transcription of OCT4 gene via aryl hydrocarbon receptor (AhR)-mediated mechanism^20^. Thus, we first attempted the ITE + Akti-1/2 combination treatment for adherent U87 cells. Compared to Akti-1/2, the ITE + Akti-1/2 combo did not have any improved effects, determined by both morphological observation (Fig. S2A) and growth curve (Fig. S2B). This is probably due to the blockage of nuclear translocation of the AhR caused by the non-specific effect of Akti-1/2^21^. Thus, Akti-1/2 is likely to offset the efficacies of ITE, and the overall effects of the ITE + Akti-1/2 combo are equivalent to that of Akti-1/2 alone.

**Figure 3:**
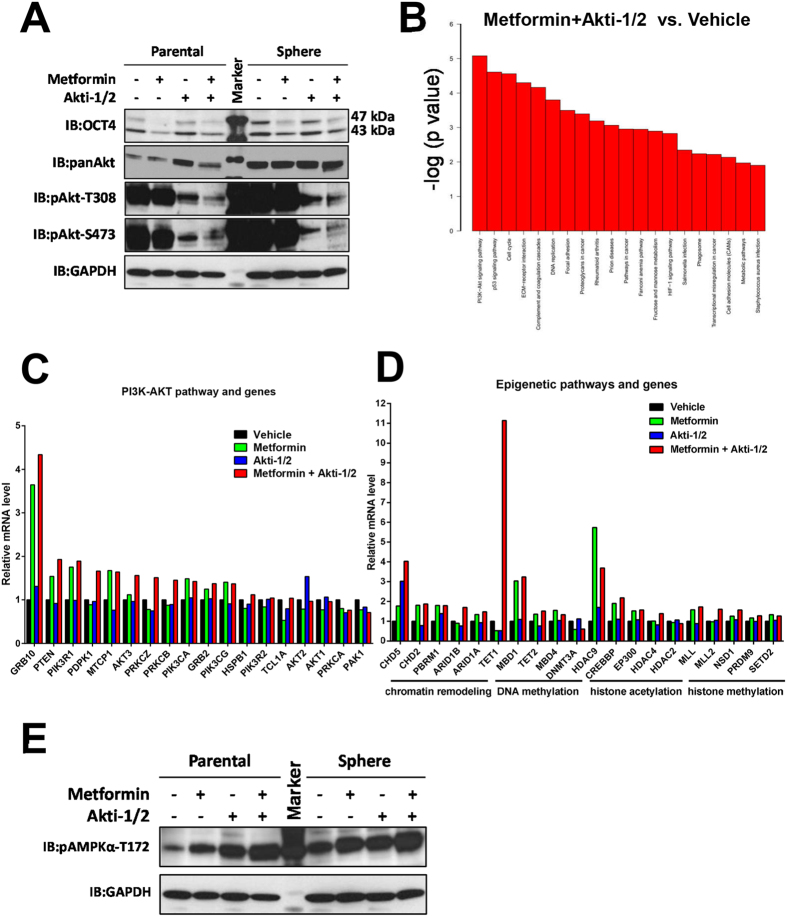
Metformin + Akti-1/2 combo alters multiple signaling and epigenetic pathways. (**A**) Adherent parental U87 cells (Parental) or U87 tumor sphere cells (Sphere) were treated with DMSO, 10 mM metformin, 5 μM Akti-1/2, or 10 mM metformin + 5 μM Akti-1/2, for 5 days. Cells were harvested and lysed, and the whole cell lysates were subjected to immunoblotting with the indicated antibodies. **(B)** U87 tumor sphere cells were treated with DMSO (Vehicle), 10 mM metformin (Metformin), 5 μM Akti-1/2 (Akti-1/2), or 10 mM metformin + 5 μM Akti-1/2 (Metformin + Akti-1/2), for 5 days. Samples were subjected to DNA microarray analyses, and the pathways most significantly affected between the “Metformin + Akti-1/2” group and the “Vehicle” group were ranked according to the p values. **(C)** Comparison of relative mRNA levels of multiple PI3K-AKT pathway genes among four groups of U87 tumor sphere cells, based on DNA microarray result in (**B**). **(D)** Comparison of relative mRNA levels of multiple epigenetic pathway genes among four groups of U87 tumor sphere cells, based on DNA microarray result in (**B**). **(E)** U87 cells treated as in (**A**) were harvested and lysed, and the whole cell lysates were subjected to immunoblotting with the indicated antibodies.

Metformin, a widely-used Type II diabetic treatment drug, was also reported to down-regulate OCT4 expression in human breast cancer cells^22^ and pancreatic cancer cells^23^. Therefore, we determined the effects of metformin + Akti-1/2 combo next. We first determined the optimal dosages of the metformin + Akti-1/2 combo against U87 cells. Treating U87 cells with serial diluted metformin + Akti-1/2 combo for 3 days resulted in varying degree of cell growth inhibition, and the estimated IC50 value of the combo against U87 was approximately 1.5 mM metformin + 1 μM Akti-1/2 (Fig. S3A). In contrast, the estimated IC50 value of the combo against Jurkat T cell-derived JLTRG cells was significantly higher (approximately 4.6 mM metformin + 4.3 μM Akti-1/2 (Fig. S3B), indicating that the combo may preferentially halt the propagation of malignant cancer cells. We chose 10 mM metformin + 5 μM Akti-1/2 for all the following studies because at this combined drug concentration the propagation of U87 cells was almost completely inhibited while that of JLTRG cells was only reduced by half. Next, we examined the effect of the combo treatment on OCT4 and AKT protein levels. As expected, metformin, by itself or in combination with Akti-1/2, significantly reduced the OCT4 protein (mainly the 47 kDa OCT4) levels in adherent parental U87 cells, and to a lesser extent, in CSC-enriched tumor sphere cells derived from the parental U87 cells (Fig. 3A). Akti-1/2 dramatically while metformin only marginally attenuated the two activated forms of AKT (pAKT-T308, pAKT-S473) in both parental and tumor sphere cells, and metformin + Akti-1/2 combo had an additive effect (Fig. 3A).

Consistent with the above immunoblotting results, global gene expression profiling of U87 tumor spheres treated with vehicle, metformin, Akti-1/2, or metformin + Akti-1/2 combo identified the PI3K-AKT signaling pathway being the most affected pathway in metformin + Akti-1/2 combo-treated sphere cells, followed by p53 signaling pathway and cell cycle pathway (Fig. 3B). Notably, compared to single treatment with either Akti-1/2 (Fig. S4A) or metformin (Fig. S4B), the combo treatment appeared to synergistically affect the PI3K-AKT pathway. In this pathway, several genes (GRB10, PTEN) were particularly affected (Fig. 3C). Since AKT is a master epigenetic modulator^9^ and OCT4 is a master epigenetic mediator^8^, the effects of combo treatment on various epigenetic aspects were examined. A number of genes involved in chromatin remodeling (e.g., ARID1B, CHD5), DNA methylation (TET1), histone methylation (MLL2, NSD1), histone acetylation (HDAC4) were significantly affected by the combo treatment (Fig. 3D). Remarkably, TET1 transcription was up-regulated by greater than 10 fold with the combo treatment (Fig. 3D), and further analysis revealed that the corresponding expression levels of several TET1/2 transcription factors were also significantly altered (Fig. S5A), confirming TET1 as one of the major effector genes in responding to the metformin + Akti-1/2 combo treatment.

Metformin is known to activate AMPK while inhibit PI3K/AKT^24^. Our immunoblotting result with U87 cells confirmed this notion and further revealed a potentiation effect of Akti-1/2 on AMPK activation (Fig. 3E). Thus, the combo treatment appeared to have synergistic effects on inhibiting AKT activation while activating AMPK due to a cross-talk between the two crucial metabolism-associated kinases. Accordingly, the combo treatment profoundly altered the expression profiles of many metabolic pathways and genes that are coordinately regulated by AKT and AMPK (Fig. S5B). In addition, it also led to the alteration of cell cycle pathways (Fig. S6A) and apoptosis pathways (Fig. S6B).

### Metformin + Akti-1/2 combo potently suppresses the propagation of U87 cells and their tumor spheres

**Figure 4:**
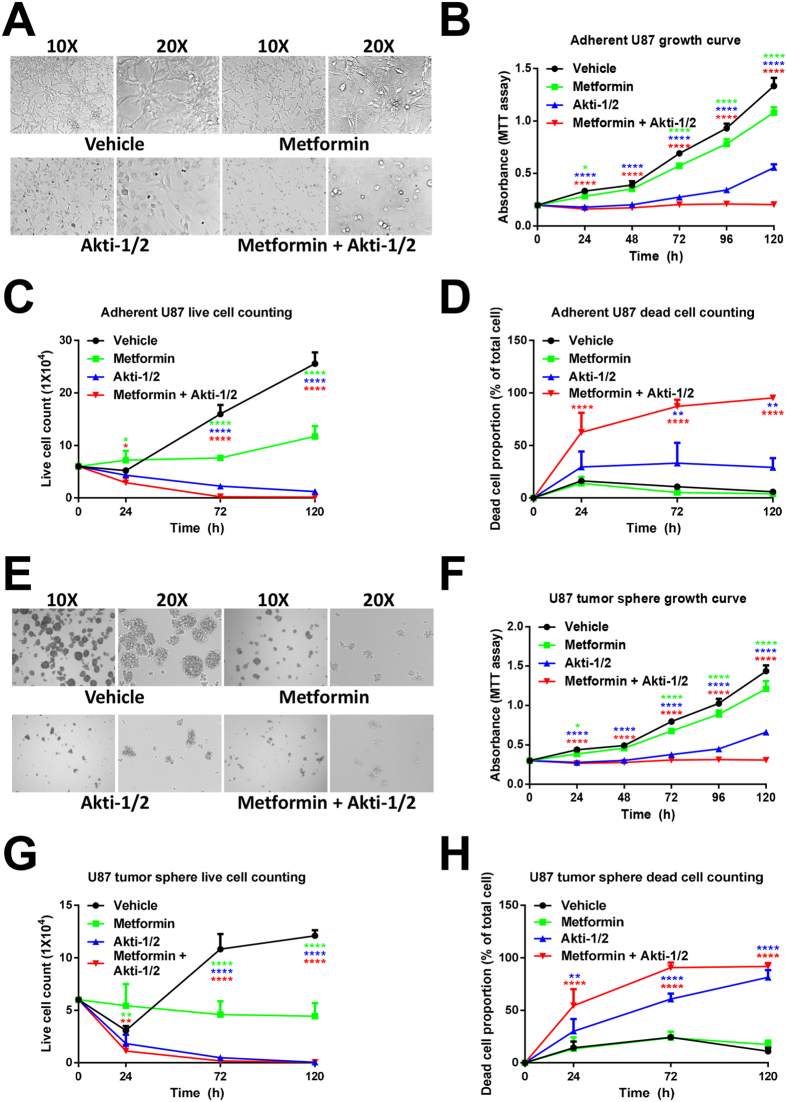
Metformin + Akti-1/2 combo potently suppresses the propagation of adherent U87 cells and their tumor spheres. **(A,E)** Adherent parental U87 cells (**A**) or U87 tumor sphere cells (**E**) were treated with DMSO (Vehicle), 10 mM metformin (Metformin), 5 μM Akti-1/2 (Akti-1/2), or 10 mM metformin + 5 μM Akti-1/2 (Metformin + Akti-1/2), for 5 days. The images were captured under an Olympus IX81 microscope at 100X (10X objective lens) or 200X (20X objective lens) magnification. **(B,F)** Adherent parental U87 cells (**B**) or U87 tumor sphere cells (**F**) were treated with the same manner as in (**A,E**) for up to 5 days. Samples were subjected to MTT assay at each time point. (**C,D,G,H**) Adherent parental U87 cells (**C,D**) or U87 tumor sphere cells (**G,H**) were treated with the same manner as in (**A,E**) for up to 5 days. Cells were collected at each time point and suspended with 100 μl PBS. Live and dead cells were counted by trypan blue exclusion using the Countess™ II FL Automated Cell Counter. Data were expressed as mean ± SD of triplicate measurements from one of three independent experiments which gave similar results. The colored asterisks indicate the difference between each treatment group and vehicle group by significance levels (*p < 0.05, **p < 0.01, ***p < 0.001, ****p < 0.0001).

Either Akti-1/2 or metformin led to certain morphological changes of adherent U87 cells (Fig. 4A) and decreases in their propagation as determined by MTT assay, with Akti-1/2 having more inhibitory effect on cell propagation (Fig. 4B). The combo treatment caused dramatic cell shrinkage, round up and detachment indicative of cell death (Fig. 4A), and almost completely blocked their propagation up to 5 days (Fig. 4B). To eliminate possible interference to the MTT assay caused by altered metabolic enzyme activities in those treated cells, we performed both manual cell counting under microscope (Fig. S7) and trypan blue exclusion-based live/dead cell counting using the Countess™ II FL Automated Cell Counter (Figs 4C, D and S8). Metformin alone blocked the increase in live cells (Fig. 4C) but did not induce cell death (Fig. 4D). Cell cycle analysis revealed that it induced a slight cell cycle arrest at the G1 phase (Fig. S9). In comparison, Akti-1/2 alone significantly reduced the live cell numbers (Fig. 4C) while increasing the proportions of dead cells (Fig. 4D) and cells arrested at the G2/M phase (Fig. S9). Remarkably, 5 days post treatment, the metformin + Akti-1/2 combo inhibited the expansion of live cells by greater than 99.5% (Fig. 4C) and greater than 95% of the cells in this group were dead (Fig. 4D). At an earlier point (2 days post treatment), the combo treatment dramatically increased the proportion of G2/M phase cells from 7% to 41% (Fig. S9). In a similar fashion, Akti-1/2 alone had much more prominent effects than metformin alone in reducing the sizes and the numbers of U87 tumor spheres enriched in CSCs (Fig. 4E), in inhibiting the propagation of tumor sphere cells (Fig. 4F), in decreasing the live sphere cell counts (Fig. 4G) and in raising the dead sphere cell proportions (Fig. 4H), and the combo had even greater effects (Fig. 4E–H).

### Metformin + Akti-1/2 combo potently attenuates the tumorigenicity of U87 cells

**Figure 5:**
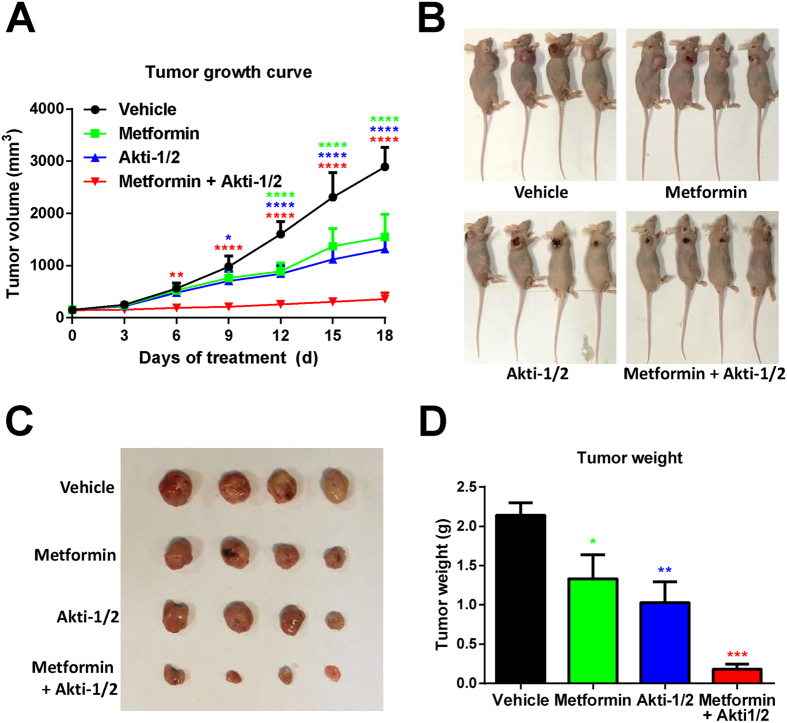
Metformin + Akti-1/2 combo potently suppresses the tumorigenicity of U87 cells. (**A**) U87 cells were inoculated subcutaneously into 16 nude mice. After tumor formation, the mice were randomly grouped and administered intratumorally with DMSO (Vehicle), 250 mg/kg/d metformin (Metformin), 50 mg/kg/d Akti-1/2 (Akti-1/2) or 250 mg/kg/d metformin + 50 mg/kg/d Akti-1/2 (Metformin + Akti-1/2), for 18 consecutive days. The averaged tumor volumes of 4 mice in each group were calculated every 3 days. **(B–D)** The sacrificed mice (**B**), the excised tumors (**C**), and the tumor weights (**D**) were shown, respectively. The data in (**A,D**) were expressed as mean ± SD of 4 mice for each group. The colored asterisks indicate the difference between each treatment group and vehicle group by significance levels (*p < 0.05, **p < 0.01, ***p < 0.001, ****p < 0.0001).

To examine the effect of combo treatment in an *in vivo* setting, U87 cells were inoculated subcutaneously into nude mice. When the xenografted tumors reached relatively small volumes (approximately 100 mm^3^), the vehicle (DMSO), metformin, Akti-1/2, or metformin + Akti-1/2 combo was administered intratumorally for twenty consecutive days, immediately followed by tumor excision and analyses. Although Akti-1/2 or metformin alone significantly reduced the tumor volumes (Fig. 5A–C) and tumor weights (Fig. 5D), the combo treatment clearly had synergistic effects (Fig. 5A–D). Thus, the metformin + Akti-1/2 combo treatment potently attenuated the tumorigenicity of U87 cells.

## Discussion

The PI3K-AKT signaling pathway comprehensively regulates cell survival, proliferation, metabolism, and stemness^10^,^11^. Its acute activation in normal stem cells can lead to senescence or depletion of the stem cell pool^25^,^26^, suggesting that it is tightly regulated in stem cell homeostasis. This pathway is usually over-activated in cancer cells^27^ and CSCs^11^,^28^, and has been widely considered as one of the major anticancer targets. In contrast, although a large body of research has documented the detection of OCT4 in cancer cells and tissues and has indicated its enrichment CSCs, considerable uncertainties and controversies still remain^12^, and only a few studies have been reported trying to directly targeting OCT4^20^,^29^. Interestingly, evidence is emerging that there exists a complicated regulatory network between OCT4 and AKT in pluripotent stem cells^13^ and CSCs^17^,^30^,^31^. On one hand, knocking-down OCT4 in embryonal carcinoma cells increased the levels of AKT1 mRNA, pAKT-T308 and pAKT-S473^17^, and reversely, inhibiting the PI3K/AKT pathway increased OCT4 expression in glioblastoma CSCs^32^. These results indicate a reciprocal negative regulation between AKT and OCT4. However, on the other hand, PI3K-AKT-activated disassociation of a transcription repressor from the OCT4 promoter was considered to account for valproic acid-induced up-regulation of OCT4 expression in mouse myoblast C2C12 cells and mouse embryonic carcinoma P19 cells^33^, and knocking-down OCT4 in pancreatic cancer cells decreased the mRNA and protein levels of total AKT^34^, implicating a positive correlation between AKT and OCT4. Such apparent discrepancy may be explained by the fact that either AKT or OCT4 controls numerous downstream targets that may indirectly regulate its counterpart in different modes at multiple levels (e.g., transcriptional, post-transcriptional, and/or post-translational level)^13^. Thus, depending on the cellular contexts, inhibiting OCT4 or AKT alone may either activate or inactivate its counterpart, resulting in considerable uncertainties in therapeutic outcomes. This led us to propose and attempt a strategy to dual inhibiting OCT4 and AKT simultaneously. Although sh-OCT4 can only partially silence OCT4 expression, by using sh-OCT4 and Akti-1/2, we provided evidence in this study that dual inhibiting OCT4 and AKT can effectively dampen the propagation of embryonal carcinoma cells, adherent cancer cells and stem-like cancer cells. We anticipate that, when combined with Akti-1/2, CRISPR/Cas9-based OCT4 knockout may reach a higher degree of inhibition on cell propagation than sh-OCT4. Taken together, we established an important proof-of-concept that dual inhibiting OCT4 and AKT can effectively target CSCs as well as the entire bulk of cancer cells.

Notably, compared with sh-OCT4 + Akti-1/2, the metformin + Akti-1/2 combo appeared to have an even higher degree of inhibition. Although metformin reduced OCT4 protein levels to some degree, it clearly functions via additional mechanisms. A well-established role of metformin is to activate the cellular metabolic sensor AMP-activated protein kinase (AMPK) and enhance the proportion of phosphorylated (i.e., activated) AMPK^24^,^35^. Since there is a reciprocal inhibition between phosphorylated AMPK and phosphorylated AKT^36^,^37^, it can be predicted that inhibiting AKT with Akti-1/2 while activating AMPK with metformin may further reduce the proportion of phosphorylated AKT while enhance that of phosphorylated AMPK, and our result was well consistent with such a prediction. Thus, the combo treatment led to a dramatically increased AMPK activation accompanied with much reduced AKT activities. However, at certain stages of cancer progression, and for some types of cancers, AMPK inhibition rather than activation may represent a potential way of therapeutic intervention, and therefore caution should be exercised in aiming at merely activating AMPK in cancer prevention and chemotherapy^38^. Importantly, the metformin + Akti-1/2 combo potently inhibited the propagation of both CSCs and differentiated cancer cells. Recent studies have demonstrated CSC’s high level of plasticity and heterogeneity, and the dynamic interconversion between CSCs and differentiated cancer cells^3^,^8^. This indicates that any successful therapeutic agents or combination of anticancer drugs must eliminate not only CSCs, but also the entire bulk of differentiated cancer cells. Thus, future study should be directed at identifying more specific and potent OCT4 inhibitors that can be used in combination with Akti-1/2 or other Akt inhibitors to target CSCs as well as differentiated cancer cells.

For molecularly-targeted therapies currently available in clinic or clinical trials, epigenetic modulators (e.g., RAS/RAF/MEK/ERK, MET, mTOR, PI3K, AKT, STAT3) and their upstream cell surface receptors (EGFR, VEGFR, IGFR) are the most common drug targets. However, when used as single agents, inhibitors targeting the above epigenetic modulators and their cell surface receptors did not yield satisfactory clinical outcomes^39^,^40^. In most cases, inhibiting the targeted components results in compensatory activation of their upstream regulators or parallel signaling pathways^41^,^42^,^43^. Thus, combinations of targeted treatments have been employed to circumvent such problems in order to yield clinical benefits^43^,^44^. Thus far, the predominant drug combos target a combination of two epigenetic modulators (and their cell surface receptors), but the relatively narrow therapeutic index of each drug plus the overlapping toxicities impose a major challenge^44^. Recently, the drug combos targeting an epigenetic modulator and an epigenetic modifier^45^, or an epigenetic modifier and the cell death pathway^46^, were reported. Our current study represents the first piece of work that simultaneously targets an epigenetic mediator and an epigenetic modulator in cancer cells (Fig. S1).

Taken together, we demonstrate here that a combination therapy simultaneously targeting an epigenetic mediator (OCT4) and an epigenetic modulator (AKT), can have significantly improved efficacies over single treatment in suppressing the propagation of CSCs as well as the entire bulk of differentiated cancer cells. Such a strategy may assist in tackling the major challenges in treating human cancers.

## Materials and Methods

### Cell lines and culture

NCCIT, U87, MCF-7 and 293T cells were purchased from American Type Culture Collection (ATCC, Rockville, USA). U251 cells were purchased from Shanghai Bogoo Biotechnology. Co., Ltd. All cells were cultured at 37 °C in DMEM (21063-029, Invitrogen, California, USA) supplemented with 10% fetal bovine serum (GIBCO 10099 or Pufei 1101-500) in a humidified 5% CO_2_ incubator (3111, Thermo Fisher Scientific, Massachusetts, USA). The details of tumor sphere culture and analysis were described previously^20^.

### Reagents and antibodies

B-27 supplement minus Vitamin A (12587-010) and basic fibroblast growth factor (bFGF) (PHG0266) were obtained from Gibco (California, USA). Epidermal growth factor (EGF) (E5036), leukemia inhibitory factor (LIF) (L5283) and DMSO (D5879) were from Sigma-Aldrich (Missouri, USA). The anti-Caspase 3 (35/17 kDa) (AC030), anti-Caspase 3 (19/17 kDa) (AC033), anti-Bax (AB026), anti-pAMPKα-T172 (AA393), and Propidium Iodide (ST511) and the Cell Cycle and Apoptosis Analysis Kit (C1052) were from Beyotime Biotechnology (Shanghai, China). The sources of other reagents were described previously^17^.

### Western blot analysis

Cells were lysed, and the whole cell lysates were boiled with SDS-PAGE sample loading buffer, separated by SDS-PAGE, blotted onto PVDF membranes as described previously^20^. The signals were visualized using the Immobilon Western Chemiluminescent HRP Substrate (Millipore WBKLS0100). In most cases, only cropped immunoblots were presented in the main figures, and the corresponding uncropped immunoblots were presented in the Supplementary Information.

### Lentiviral vector construction, viral production and viral infection

Lentiviral vector construction and viral production were carried out as previously described^17^. For viral infection, cells were cultured overnight, and the culture media were replaced with viral supernatants supplemented with 8 μg/ml of polybrene (AL-118, Sigma-Aldrich, USA). The multiplicity of infection (MOI) was estimated to be between 0.5 to 2. 8–10 h later, the viral supernatants were replaced with fresh culture medium to allow further growth until use.

### Cell propagation, cell viability and cell cycle assays

Cell propagation was determined with the Cell Proliferation Kit I (MTT) (Roche 11465007001) according to the manufacturer’s instructions. Briefly, cells grown in 96-well plates (5,000–10,000 cells/well) or 6-well plates (2 × 10^5^–4 × 10^5^ cells/well) were treated with DMSO (Vehicle), 10 mM metformin (Metformin), 5 μM Akti-1/2 (Akti-1/2) or 10 mM metformin + 5 μM Akti-1/2 (Metformin + Akti-1/2) for specified durations. 10 μl of the MTT labeling reagent (final concentration 0.5 mg/ml) was added into each well, and the plate was incubated for 4 h at 37 °C with 5% CO_2_. The absorbance was measured using a microplate reader (Beckman Coulter DTX880) at the wavelength of 570 nm.

For live/dead cell counting, U87 cells grown in 24-well plates (60,000 cells/well) in DMEM or NSC medium were treated with DMSO or reagents for specified durations. Cells were collected by centrifugation at 1000 *g* for 2 min and resuspended with 100 μl PBS. The live and dead cells were counted by trypan blue exclusion method using the Countess™ II FL Automated Cell Counter.

For cell cycle analysis, U87 cells grown in 6-well plates (200,000 cells/well) in DMEM medium were treated with DMSO or reagents for 48 h. Cells were collected by centrifugation at 1000 *g* for 5 min, washed twice with ice-cold PBS, fixed with 70% ice-cold ethanol and stored at 4 °C for 24 h. Cells were then washed with cold PBS and stained with 0.5 ml of propidium iodide (PI) staining buffer contained in the Cell Cycle and Apoptosis Analysis Kit at 37 °C for 30 min in the dark. Flow cytometric analysis of cell cycle was performed with the Guava^®^ easyCyte flow cytometer.

### Mouse xenograft tumor models

BALB/c nude mice (female, 3–4 weeks old) were purchased from Shanghai Experimental Animal Centre, Chinese Academy of Science. Experimental animals were kept in the central animal facility of the Zhejiang University School of Medicine and housed in laminar-flow cabinets under specific pathogen-free conditions with a 12 h light/dark cycle. All studies on mice were conducted in accordance with the National Institute Guide for the Care and Use of Laboratory Animal. The animal protocol has been approved by the Committee of the Ethics of Animal Experiments of Zhejiang University. Subcutaneous xenografting experiments were performed as described previously^20^. Briefly, U87 cells (2.5 × 10^6^) were inoculated subcutaneously into each mouse. When the tumor volume reached approximately 100 mm^3^, the mice were divided into homogenous blocks based on their tumor volumes followed by randomly assigning each block into the vehicle control and treatment groups (n = 4/group). The vehicle (DMSO) or treatment compounds were administered to the mice by intratumoral injection once daily for 21 consecutive days, at the dose of 250 mg metformin/kg/d, or 50 mg Akti-1/2/kg/d, or a combination of both compounds. The external diameter of the tumors was measured every other day as described previously^17^.

### Immunofluorescence microscopy

Adherent live NCCIT cells grown in 6-well plates were washed twice with PBS gently, and stained with 5 μg/ml PI for 10 min at 37 °C, followed by two gentle washes with PBS. Cells were examined using an Olympus fluorescence microscope at the excitation wavelength of 535 nm, and the red fluorescent cells that represent late phase apoptotic cells were counted in captured images.

### DNA microarray and analyses

Briefly, RNA was extracted from four groups of U87 neurosphere cells by TRIzol method according to the manufacturer’s instructions. Reverse transcription to the first-strand cDNA was primed with T7 oligo (dT) primer to synthesize cDNA containing a T7 promoter sequence. Second-strand cDNA synthesis converts the single-stranded cDNA into a double-stranded DNA (dsDNA) template for transcription. The hybridization was carried out by GeneChip Hybridization, Wash, and Stain kit (affymetrix: 900720). The arrays were scanned at 570 nm with a confocal scanner from Affymetrix. Analysis of the arrays was performed using the GeneSpring 12.6. Normalization of the array was performed using a robust multiarray analysis (RMA). A p-value cutoff of 0.05 was used to filter genes that were significantly expressed between the two samples. A fold change of greater than 2 was used as a criterion for differential expressed genes (DEGS). Clustering of DEGs was created from GeneSpring by using Pearson centered correlation and average linkage method.

The Database for Annotation, Visualization and Integrated Discovery (DAVID) has been used to assess the functional clustering of DEGs. Significant GO process terms and KEGG pathway of DEGs were identified using hypergeometric algorithms test with Benjamini & Hochberg multiple testing correction. The significant GO biological process terms were determined with the thresholds of p-value < 0.01 and enrichment gene count >2, whereas KEGG pathway enrichment analyses of the selected DEGs were determined with the thresholds of p-value < 0.05 and enrichment gene count >2.

### Statistical analysis

All statistical analyses were carried out using the GraphPad Prism 6.0 statistics software published by GraphPad Software, Inc. All quantitative data were presented as means ± SD of triplicate measurements from one of three independent experiments which gave similar results. The statistical significance was evaluated using one-way ANOVA or two-way ANOVA, and p < 0.05 was considered statistically significant.

## Additional Information

**How to cite this article**: Li, W. *et al*. Dual inhibiting OCT4 and AKT potently suppresses the propagation of human cancer cells. *Sci. Rep.* **7**, 46246; doi: 10.1038/srep46246 (2017).

**Publisher's note:** Springer Nature remains neutral with regard to jurisdictional claims in published maps and institutional affiliations.

## Supplementary Material

Supplementary Information
