# Supplementary material for: Dual inhibiting OCT4 and AKT potently suppresses the propagation of human cancer cells
Source: Sci Rep. 2017 Apr 6;7:46246. doi: 10.1038/srep46246 (PMC5382782; doi:10.1038/srep46246)
Supplement: Supplementary Information [file srep46246-s1.pdf]

Supplementary Information

Dual inhibiting OCT4 and AKT potently suppresses the propagation of human cancer cells

Wenxin Li[1,*], Yanwen Zhou[1,*], Xiaoqian Zhang[1], Ying Yang[1], Songsong Dan[1], Tong Su[2], Shiqi She[1], Weilai Dong[2], Qingwei Zhao[1,3], Jia Jia[4], Hangping Yao[1], Min Zheng[1], Bo Kang[1], Ying-Jie Wang[1]

[1]State Key Laboratory for Diagnosis and Treatment of Infectious Diseases, Collaborative Innovation Center for Diagnosis and Treatment of Infectious Diseases, the First Affiliated Hospital, School of Medicine, Zhejiang University, Hangzhou, Zhejiang 310003, China.
[2]College of Life Sciences, Zhejiang University, Hangzhou, Zhejiang 310058, China.
[3]Department of Pharmacy, the First Affiliated Hospital, School of Medicine, Zhejiang University, Hangzhou, Zhejiang 310003, China.
[4]Shanghai Center for Bioinformation Technology, Shanghai 201203, China.

*These authors contributed equally to this work.
Correspondence and requests for materials should be addressed to Y.-J.W. (email: yingjiewang@zju.edu.cn) or B.K. (email: kangbo@zju.edu.cn)

Figure S1

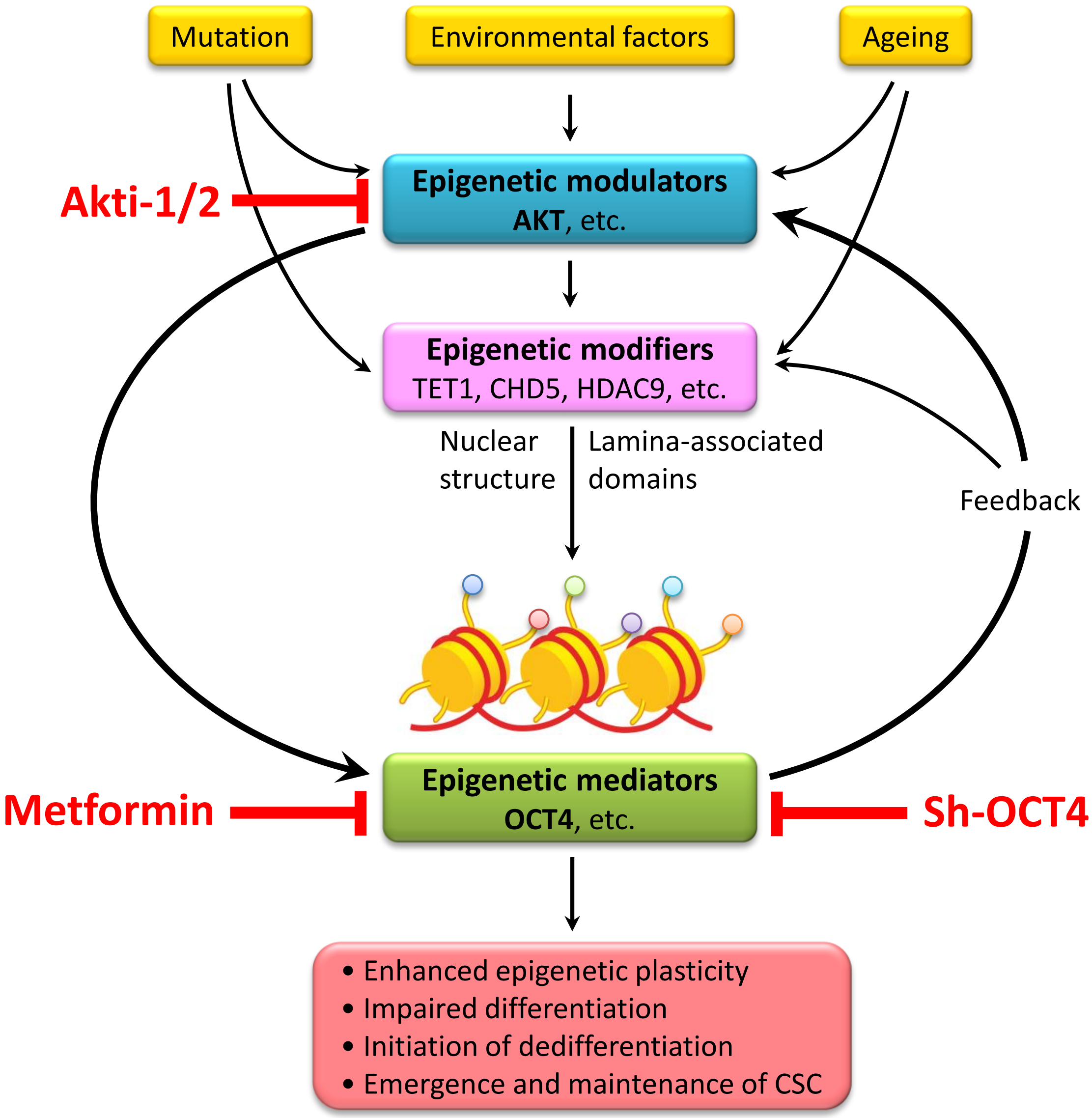

Figure S1. Graphical summary. Based on their roles in regulating cancer epigenetics and their contribution to malignancy, three classes of genes were defined: the epigenetic modulators, the epigenetic modifiers, and the epigenetic mediators. The epigenetic modulators (e.g., AKT) and the epigenetic mediators (e.g., OCT4) can regulate each other in a reciprocal manner, and inhibiting either one may activate the other. The current study demonstrated that a combination therapy simultaneously targeting an epigenetic mediator (OCT4) and an epigenetic modulator (AKT), can have significantly improved efficacies over single treatment in suppressing the proliferation of CSCs as well as the entire bulk of differentiated cancer cells. The depiction was adapted from Figure 1 in Ref. 8.

Figure S2

A

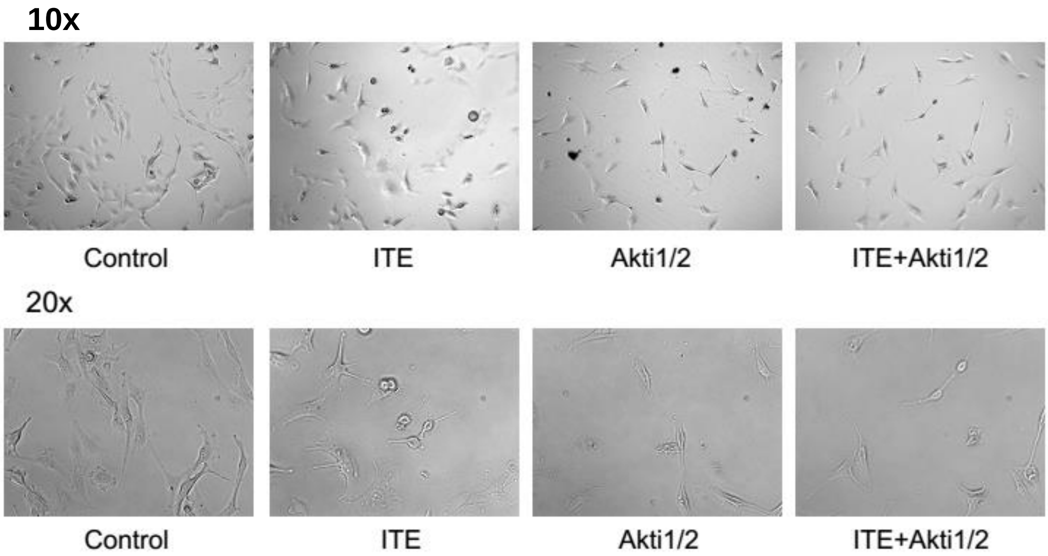

B

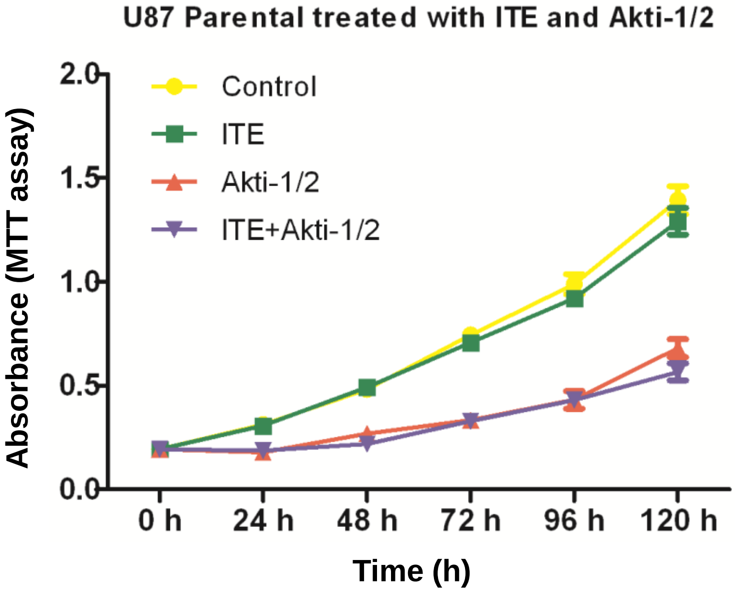

Figure S2. Effect of ITE+Akti-1/2 combo on the propagation of adherent U87 cells. (A) Adherent parental U87 cells were treated with DMSO (Control), 10 μM ITE (ITE), 5 μM Akti-1/2 (Akti-1/2), or 10 μM ITE + 5 μM Akti-1/2 (ITE + Akti-1/2), for 5 days. The images were captured under an Olympus IX81 microscope at 100× (10× objective lens) or 200× (20× objective lens) magnification. **(B)** Cells treated as in (A) were subjected to MTT assay at each time point. Data were expressed as mean ± SD of triplicate measurements from one of three independent experiments which gave similar results.

Figure S3

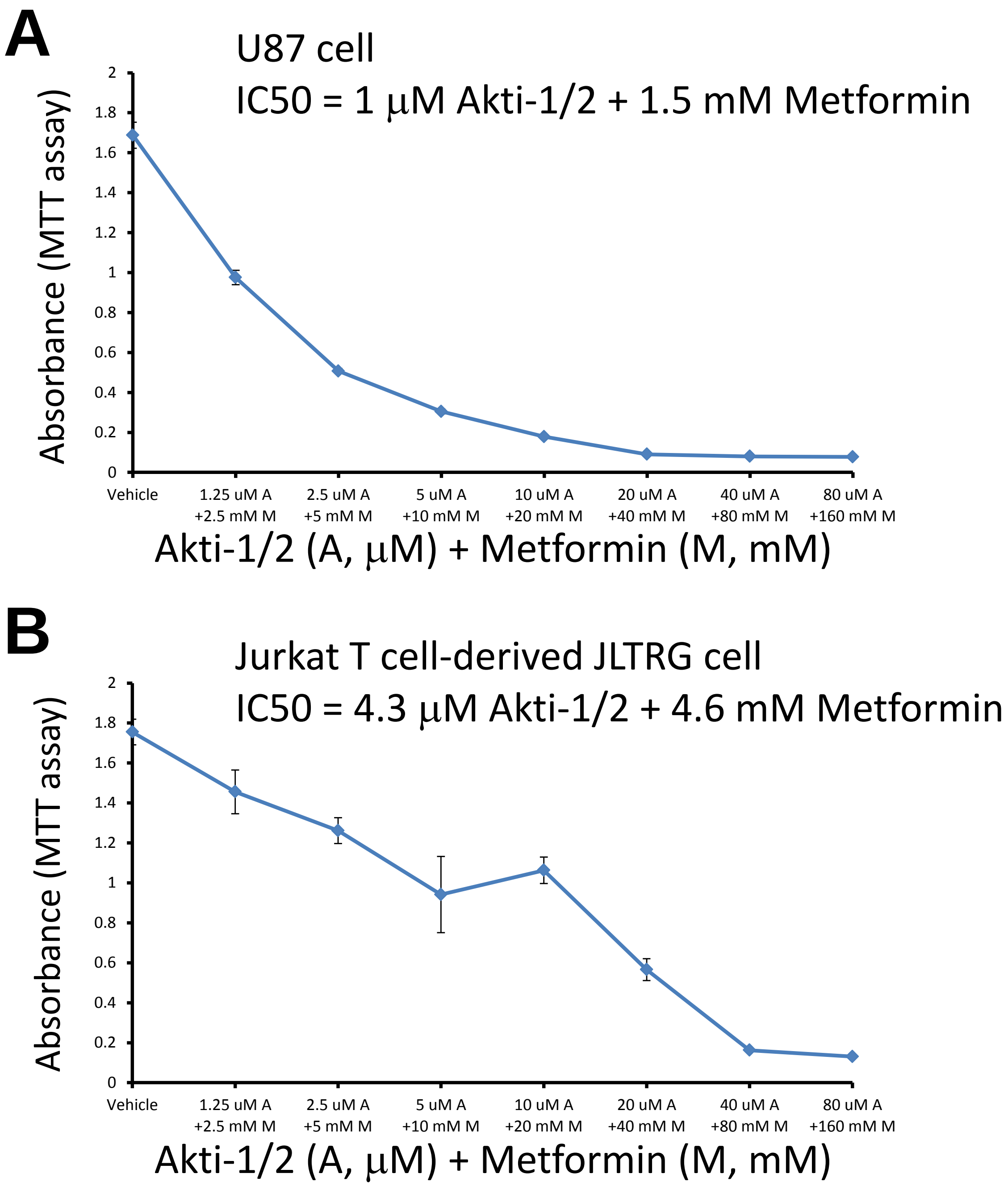

Figure S3. Dose curves of the metformin+Akti-1/2 combo against U87 cells and JLTRG cells. (A) Adherent parental U87 cells were treated with serial diluted combinations of metformin and Akti-1/2 for 3 days, and subjected to MTT assay. **(B)** Jurkat T cell-derived JLTRG cells were grown in RPMI 1640 supplemented with 10% FBS, and treated with serial diluted combinations of metformin and Akti-1/2 for 3 days, and subjected to MTT assay. Data were expressed as mean ± SD of triplicate measurements from one of two independent experiments which gave similar results.

Figure S4

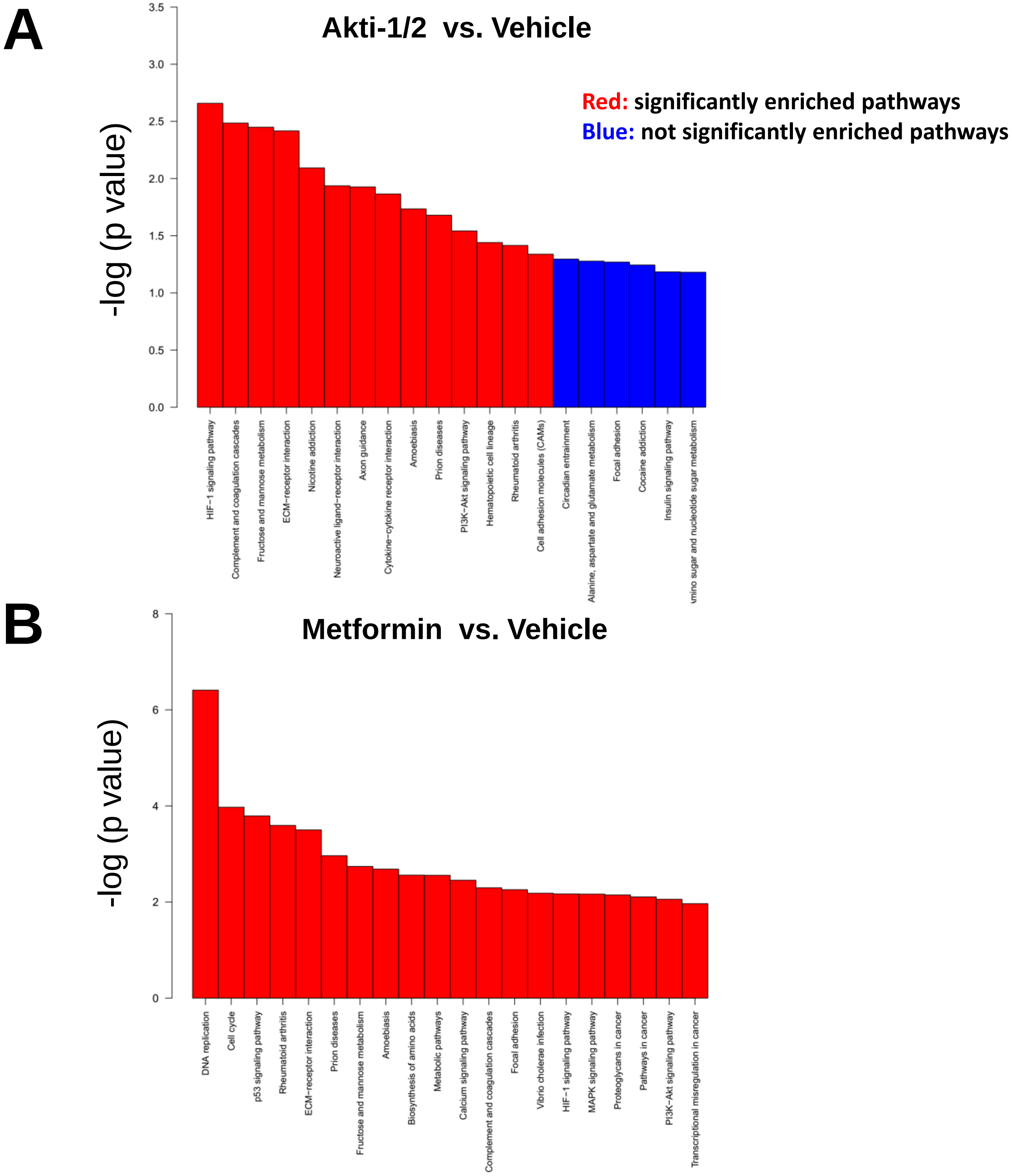

Figure S4. Metformin+Akti-1/2 combo synergistically affects the PI3K-AKT pathway. (A, B) U87 tumor sphere cells were treated with DMSO (Vehicle), 10 mM metformin (Metformin), 5 μM Akti-1/2 (Akti-1/2), or 10 mM metformin + 5 μM Akti-1/2 (Metformin + Akti-1/2), for 5 days. Samples were subjected to DNA microarray analyses, and the pathways most significantly affected between the "Akti-1/2" group and the "Vehicle" group (A), or those between the "Metformin" group and the "Vehicle" group (B) were ranked according to the p values. In contrast to Figure 3(B) where the "Metformin + Akti-1/2" group and the "Vehicle" group were compared and the PI3K-AKT pathway was by far the most affected pathway, single treatment with either Akti-1/2 or metformin alone had much less effect on this pathway.

Figure S5

A

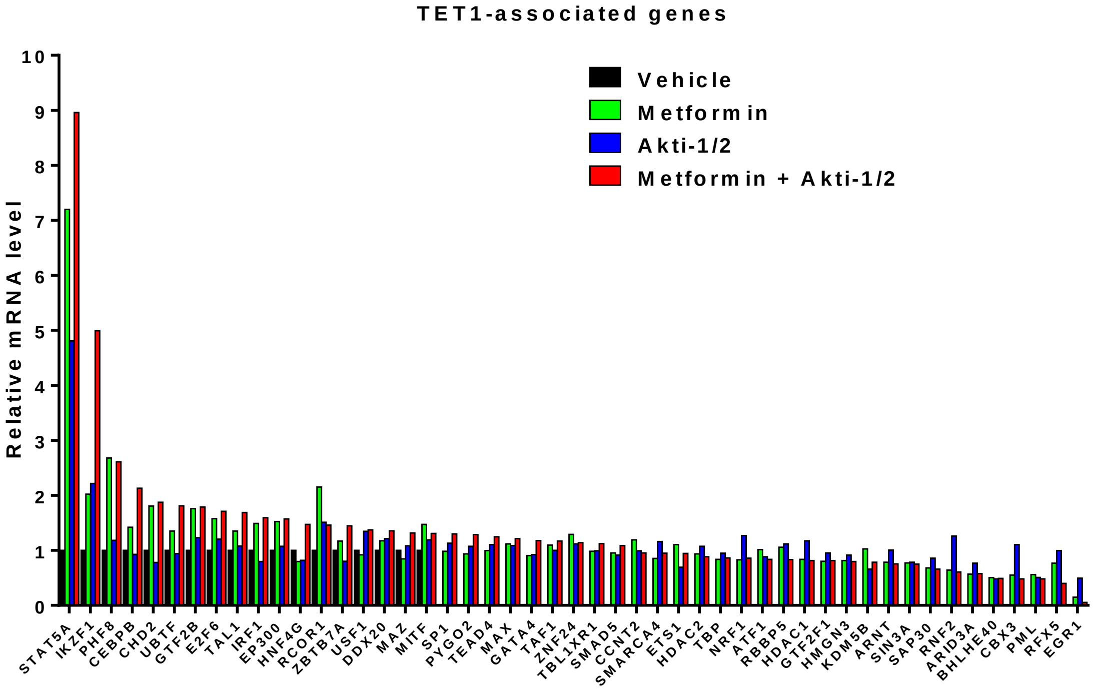

B

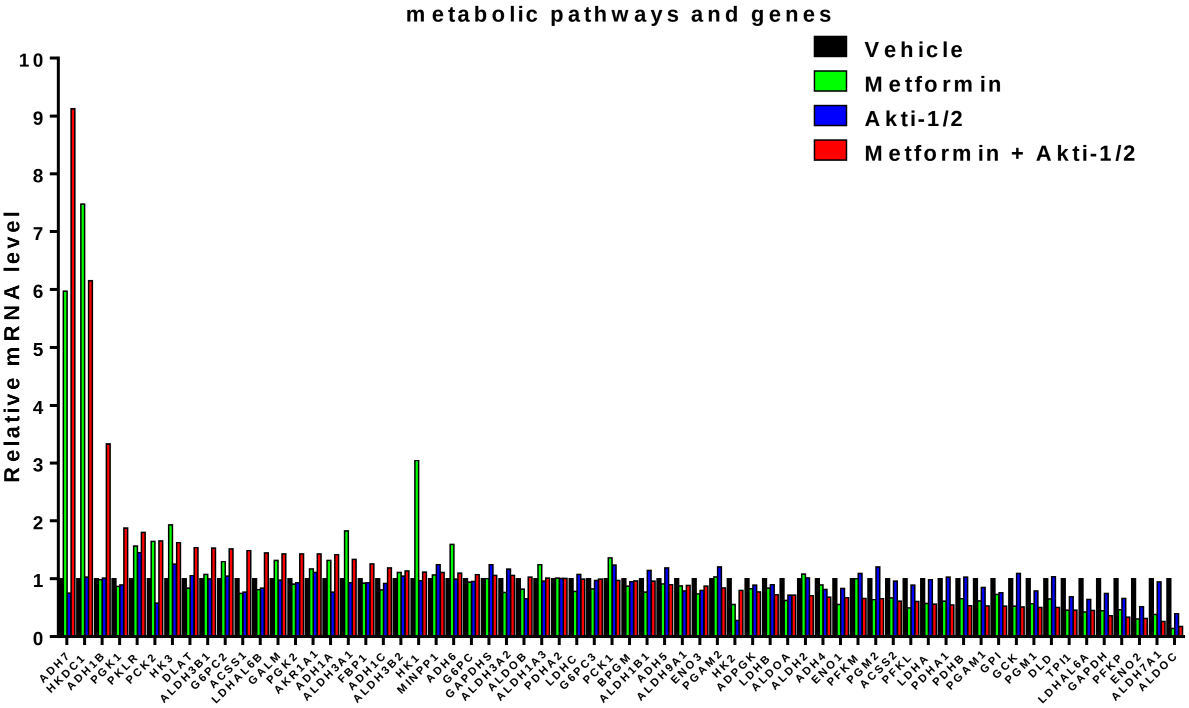

Figure S5. Metformin+Akti-1/2 combo alters TET1-associated epigenetic pathways and metabolic pathways. (A, B) U87 tumor sphere cells were treated with DMSO (Vehicle), 10 mM metformin (Metformin), 5 μM Akti-1/2 (Akti-1/2), or 10 mM metformin + 5 μM Akti-1/2 (Metformin + Akti-1/2), for 5 days. Samples were subjected to DNA microarray analyses. The relative mRNA levels of TET1-associated genes (A) or metabolic pathway associated genes (B) among the four groups were presented.

Figure S6

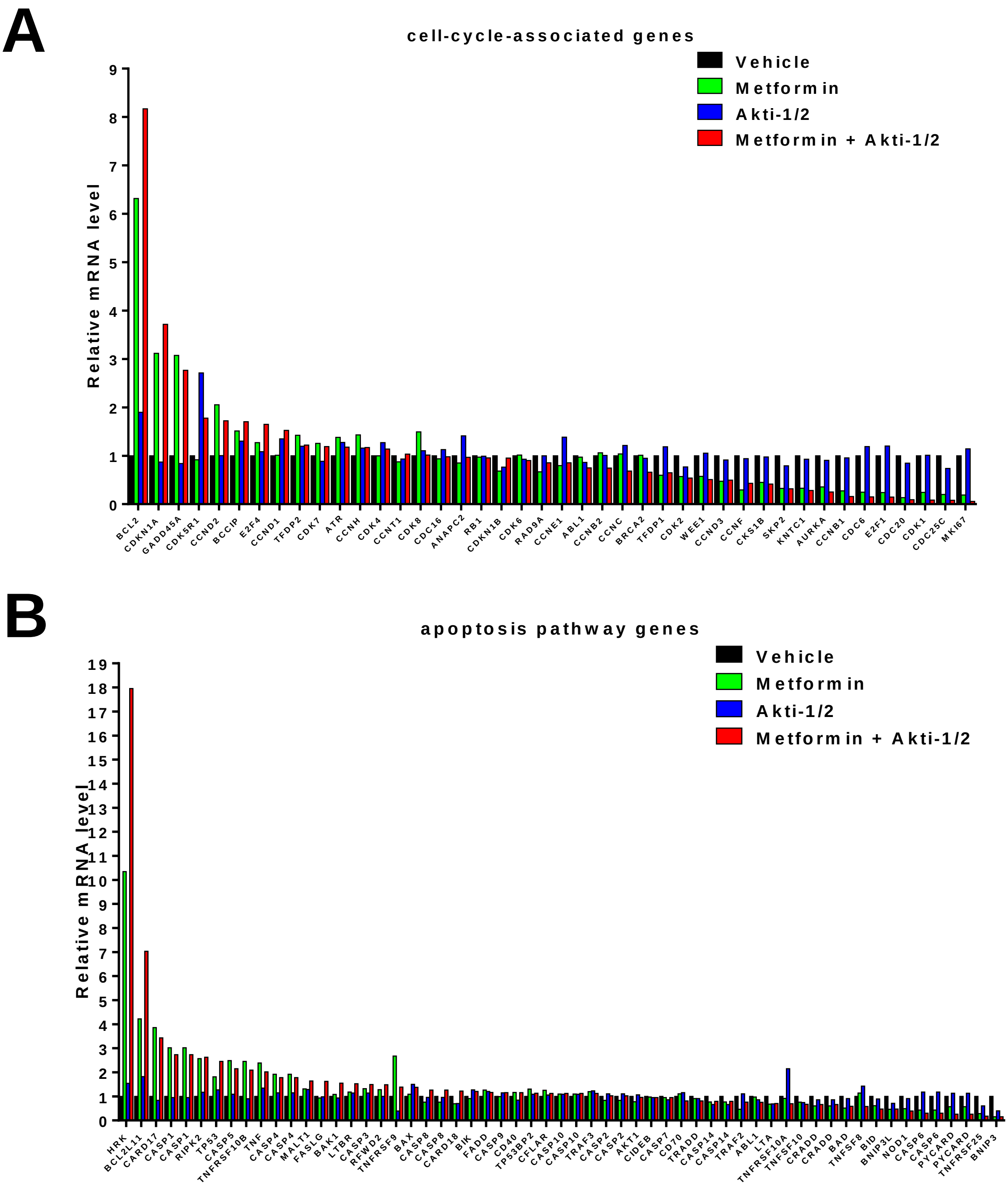

Figure S6. Metformin+Akti-1/2 combo alters cell cycle-associated and apoptosis-associated genes. (A, B) U87 tumor sphere cells were treated with DMSO (Vehicle), 10 mM metformin (Metformin), 5 μM Akti-1/2 (Akti-1/2), or 10 mM metformin + 5 μM Akti-1/2 (Metformin + Akti-1/2), for 5 days. Samples were subjected to DNA microarray analyses. The relative mRNA levels of cell cycle-associated genes (A) or apoptosis-associated genes (B) among the four groups were presented.

Figure S7

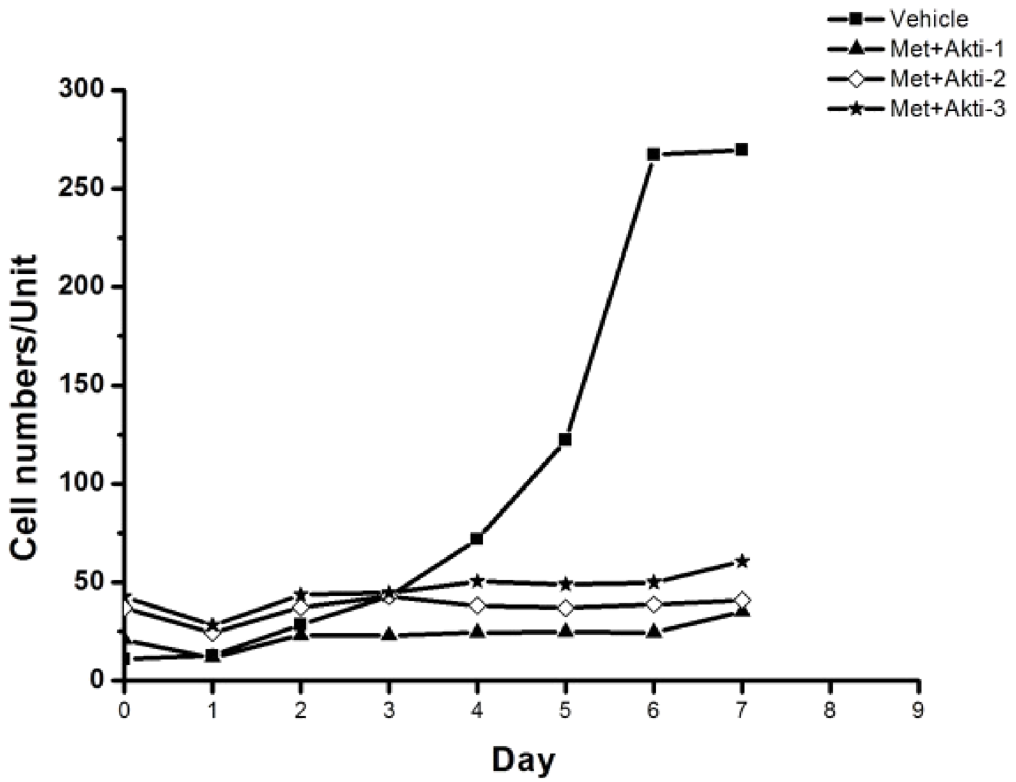

Figure S7. Metformin+Akti-1/2 combo potently suppresses the propagation of adherent U87 cells. Adherent parental U87 cells were cultured in 24-well plates, and treated with DMSO (Vehicle), or 10 mM metformin + 5 μM Akti-1/2 (Met + Akti, triplicate wells), for 5 days. Five images were captured for each well at each time point under an Olympus IX81 microscope at 40× (4× objective lens) magnification, and cell numbers were counted in a randomly chosen 200 mm × 200 mm square in each image.

Figure S8

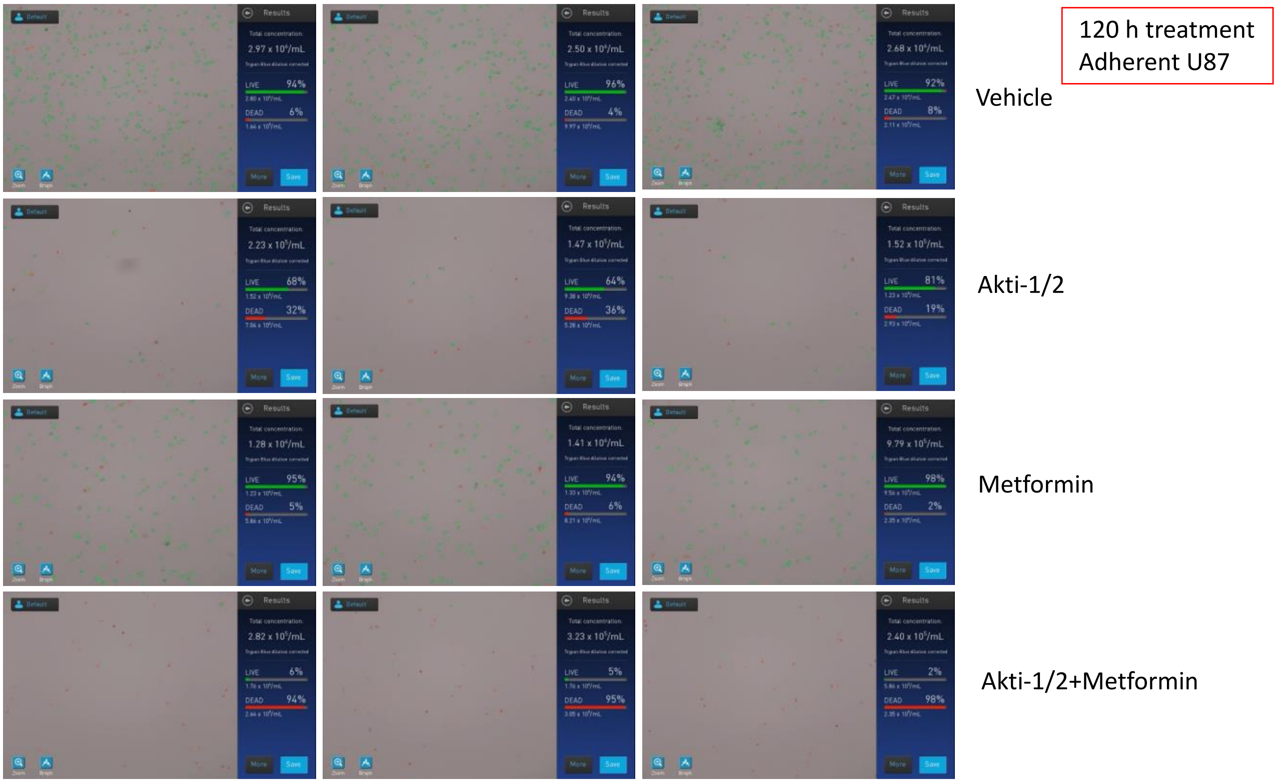

Figure S8. Metformin+Akti-1/2 combo potently induces cell death of adherent U87 cells. Adherent parental U87 cells were cultured in 24-well plates, and treated with DMSO (Vehicle), 10 mM metformin (Metformin), 5 µM Akti-1/2 (Akti-1/2), or 10 mM metformin + 5 µM Akti-1/2 (Metformin + Akti-1/2), for 5 days. Cells were collected and suspended with 100 µl PBS. Live and dead cells were counted by trypan blue exclusion using the Countess™ II FL Automated Cell Counter. The raw data of the cell counting are presented here, and the corresponding calculated data are presented in Figure 4C and 4D.

Figure S9

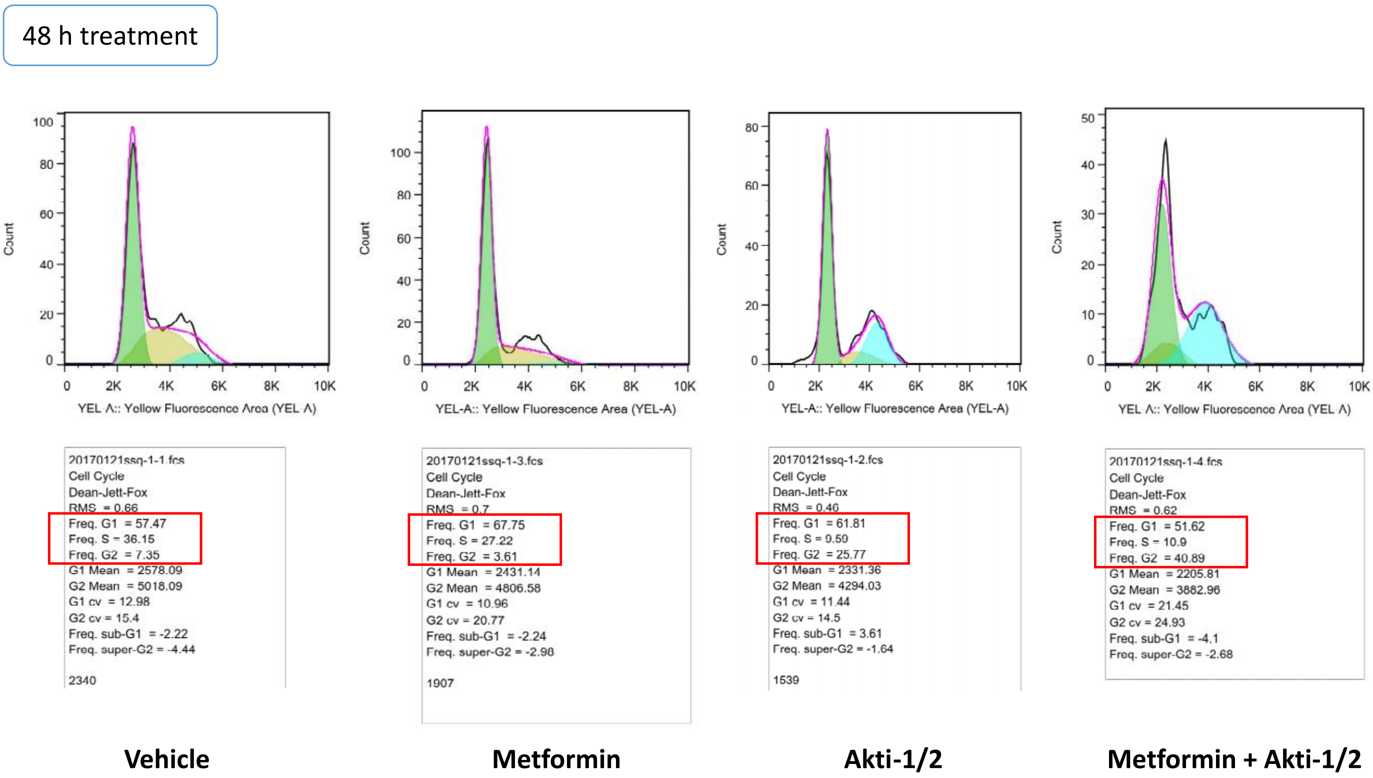

Figure S9. Metformin+Akti-1/2 combo potently induces cell cycle arrest of adherent U87 cells. Adherent parental U87 cells were cultured in 6-well plates, and treated with DMSO (Vehicle), 10 mM metformin (Metformin), 5 µM Akti-1/2 (Akti-1/2), or 10 mM metformin + 5 µM Akti-1/2 (Metformin + Akti-1/2), for 2 days. Cells were collected, washed and fixed with 70% cold ethanol, followed by being stained with propidium iodide and analyzed with flow cytometry. The percentages of cells present in G1, S, and G2/M phases of the cell cycle are highlighted with red boxes.

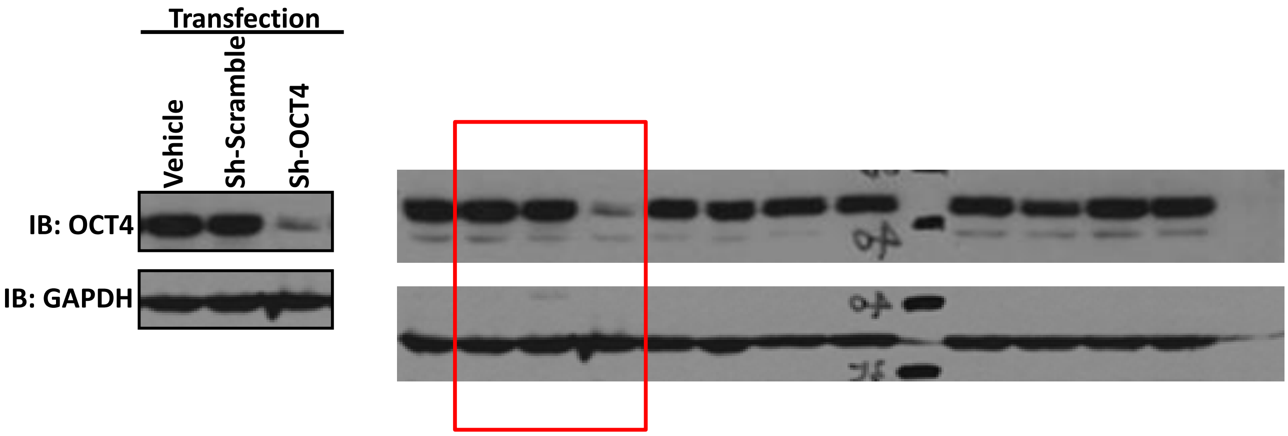

Uncropped immunoblots for Figure 1A

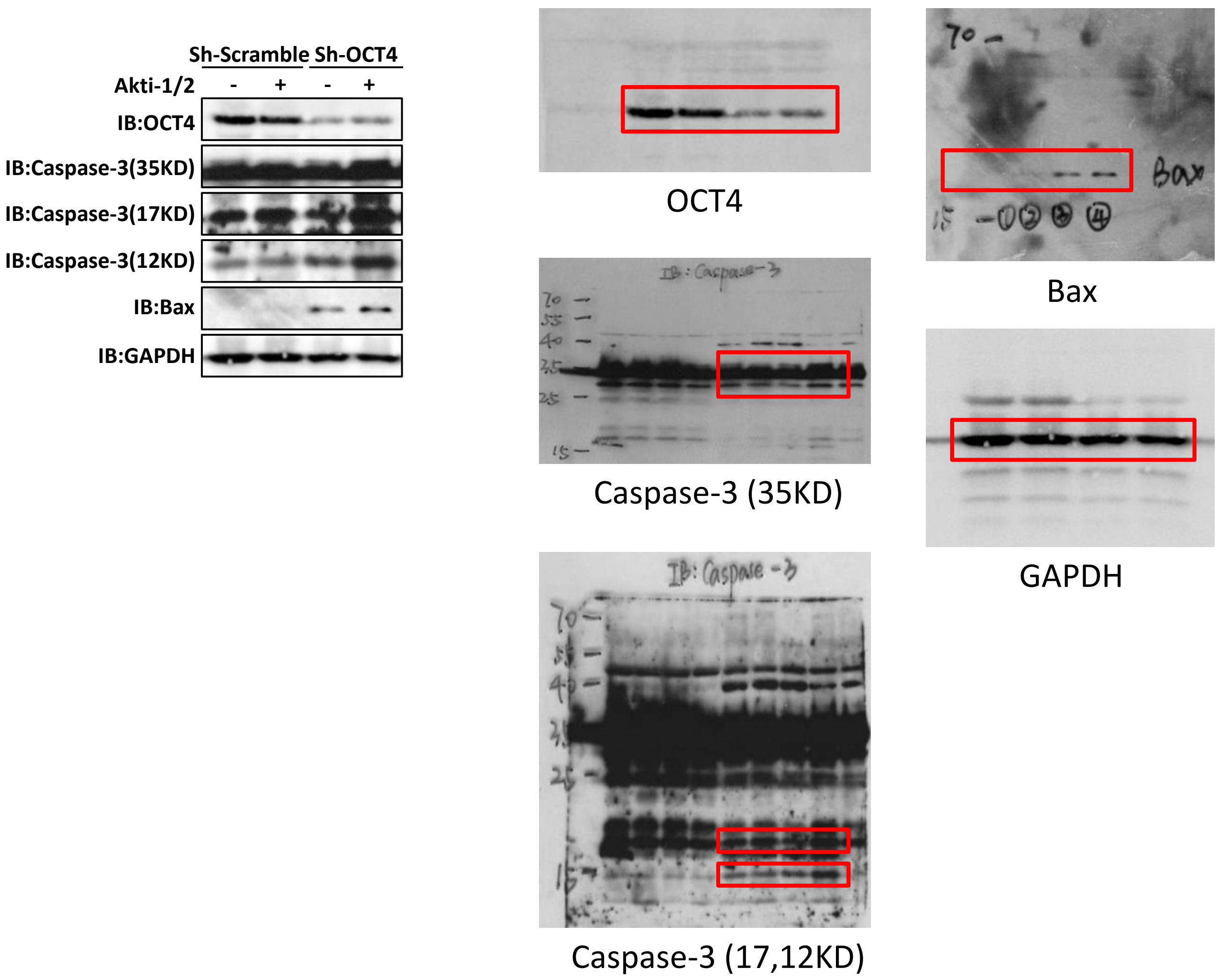

Uncropped immunoblots for Figure 1D

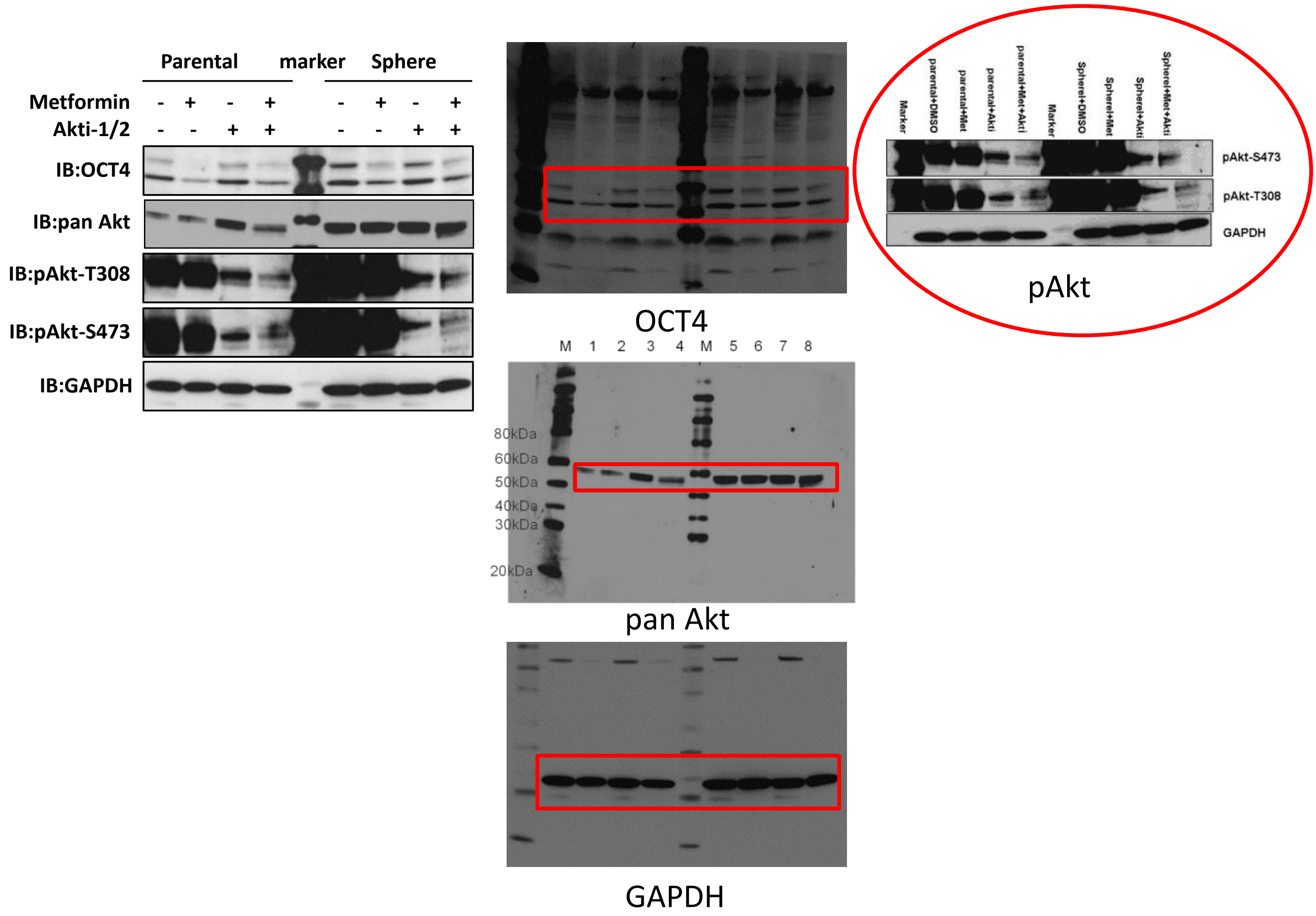

Uncropped immunoblots for Figure 3A

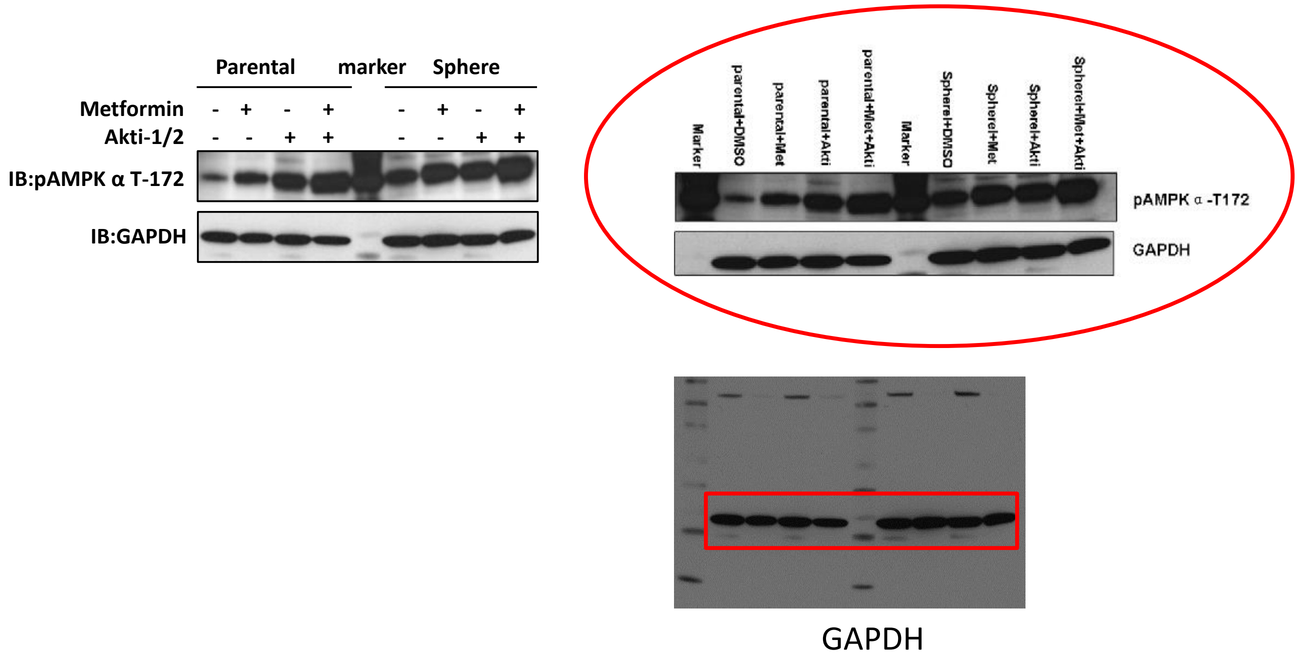

Uncropped immunoblots for Figure 3E
